# ^68^Ga-FAPI-04 PET/CT has high diagnostic efficacy in Tg-positive and Tg-negative differentiated thyroid cancer

**DOI:** 10.1371/journal.pone.0355343

**Published:** 2026-09-03

**Authors:** Lixian Mou, Ting Zhang, Xiaoling Zhang, Jian Zhou, Yue Chen, Wei Wang

**Affiliations:** 1 Department of Nuclear Medicine, Affiliated Hospital of Southwest Medical University, Luzhou, Sichuan, China; 2 Nuclear Medicine and Molecular Imaging Key Laboratory of Sichuan Province, Luzhou, Sichuan, China; 3 Laboratory for Targeted Radiopharmaceuticals Creation, Luzhou, Sichuan, China; 4 Institute of Nuclear Medicine, Southwest Medical University, Luzhou, Sichuan, China; 5 Department of General Practice, Affiliated Hospital, Southwest Medical University, Luzhou, Sichuan, China; 6 Department of Pathology, Affiliated Hospital, Southwest Medical University, Luzhou, Sichuan, China; Università degli Studi di Brescia: Universita degli Studi di Brescia, ITALY

## Abstract

**Background:**

Thyroglobulin (Tg) is a standard biomarker for monitoring differentiated thyroid carcinoma (DTC) after radioiodine therapy, yet its reliability is compromised by false-positive and false-negative results. This study aimed to evaluate the diagnostic performance of ^68^Ga-FAPI-04 PET/CT, an emerging imaging modality, for detecting recurrence and metastasis in DTC patients, regardless of their Tg status.

**Methods:**

This retrospective study included patients with suspected or confirmed metastatic DTC who underwent ^68^Ga-FAPI-04 PET/CT between October 2022 and May 2025. All participants underwent ^68^Ga-FAPI-04 PET/CT and Tg testing, with histopathology or clinical follow-up as the reference standard. The sensitivity, specificity, and accuracy of both methods were compared. Quantitative analysis was performed using the target-to-background ratio (TBR).

**Results:**

Among 99 patients in a TSH-suppressed state, ^68^Ga-FAPI-04 PET/CT demonstrated superior diagnostic accuracy (95.96%) compared to Tg testing (91.92%). In 48 TSH-stimulated patients, PET/CT maintained perfect accuracy (100%), outperforming Tg (97.92%). Notably, PET/CT successfully identified metastatic lesions in eight Tg-negative patients, primarily lymph node metastases. Using the mediastinum as a background for TBR calculation further enhanced the diagnostic efficacy for lymph node metastases, with the optimal threshold being 1.428, and achieving an accuracy and true positive rate of 0.98.

**Conclusion:**

^68^Ga‑FAPI‑04 PET/CT exhibits high accuracy for diagnosing recurrence and metastasis in DTC, and importantly can identify lesions in Tg‑negative patients.

## Introduction

Over the past 20 years, the global incidence of thyroid cancer has exhibited a consistent annual increase. In 2020, the age-standardised incidence rates of thyroid cancer were reported as 10.1 per 100 000 women and 3.1 per 100 000 men, and age-standardised mortality rates were 0.5 per 100 000 women and 0.3 per 100 000 men [1]. Differentiated thyroid carcinoma (DTC) accounts for more than 90% of all thyroid cancers [2]. Furthermore, more than 80% of participants with DTC achieve an excellent response to current treatment strategies. After total thyroidectomy with DTC, treatment with radioactive iodine-131 ^(131^I) is usually performed to remove residual thyroid and lesions [2]. Postoperative monitoring often involves measuring serum thyroglobulin (Tg) levels, which can provide critical insights into the likelihood of achieving remission or experiencing persistent or recurrent disease following initial therapy [3]. In patients who have undergone total thyroidectomy followed by ^131^I therapy, Tg level of less than 1 ng/mL after thyroid-stimulating hormone (TSH) stimulation (achieved either through medication withdrawal or recombinant human TSH (rhTSH) injection) typically indicates a low risk of disease recurrence. Conversely, a Tg level below 0.2 ng/mL during TSH suppression is regarded as the optimal clinical outcome [4,5]. However, patients are generally required to discontinue thyroid hormone therapy temporarily before evaluation, which can lead to symptoms of hypothyroidism. In addition, serum Tg levels can only reflect the active degree of the tumor and cannot locate the recurrence and metastasis [6,7].

Fibroblast-activating protein (FAP) has been recently identified to be highly expressed in cancer-associated fibroblasts (CAFs) and is closely related to cancer cell proliferation, tumor immunity, angiogenesis, extracellular matrix remodeling, and metastasis. FAP exhibits low expression levels in normal tissues and organs, rendering it an advantageous molecular target for tumor diagnosis andtreatment [8,9]. Increased FAP expression is positively correlated with the dediﬀerentiation and aggressiveness of thyroid cancer. Recent advancements in molecular imaging have highlighted the efficacy of ^68^Ga-FAPI-04 PET/CT in diagnosing various cancers, particularly those associated with the digestive system and gynecological malignancies [10–12]. This imaging modality has shown significant promise in enhancing diagnostic accuracy and influencing treatment planning.

DTC which include papillary and follicular thyroid cancer, often presents a diagnostic challenge, especially in cases where patients exhibit elevated Tg levels but negative iodine scintigraphy, a condition known as TENIS (Tg elevation, negative iodine scintigraphy) syndrome. In such scenarios, conventional imaging techniques may fail to localize recurrent or metastatic disease, necessitating the exploration of alternative imaging strategies. In a study comparing ^68^Ga-FAPI-04 PET/CT with ^18^F-FDG PET/CT, it was found that ^68^Ga-FAPI-04 PET/CT may have higher sensitivity in detecting primary tumors and involved lymph nodes in cases of DTC with TENIS. This suggests that ^68^Ga-FAPI-04 PET/CT could be particularly useful in localizing recurrent or metastatic lesions in patients with TENIS, thereby aiding in the management of this challenging condition [13]. Nevertheless, limitations in sample size and biases in data collection persist in the diagnosis of DTC using ^68^Ga-FAPI PET/CT. Clinically, instances of thyroid cancer recurrence or metastasis have been observed even in patients with negative Tg levels. For these patients, the diagnostic efficacy of ^68^Ga-FAPI PET/CT has not yet been reported. Therefore, ⁶⁸Ga-FAPI-04 PET/CT may be useful not only in patients with TENIS syndrome but also in various states of DTC, including both Tg-positive and Tg-negative conditions, regardless of TSH stimulation status. Thus, this study aims to collect data randomly from patients with DTC who have undergone surgery and radioactive ^131^I treatment to evaluate the diagnostic efficacy of ^68^Ga-FAPI PET/CT during their follow-up.

## Materials and methods

The imaging and clinical data analyzed in this retrospective study were derived from patients who underwent ^68^Ga-FAPI-04 PET/CT scans at the Affiliated Hospital of Southwest Medical University between October 2021 and May 2025. This retrospective analysis was reviewed and approved by the Ethics Committee of the Affiliated Hospital of Southwest Medical University (Approval No. AHSWMU-2020–035). This study was conducted in accordance with the ethical principles outlined in the Belmont Report and the Declaration of Helsinki. All patients had provided written informed consent for the original imaging procedure and data collection. For this retrospective study, the researchers accessed the data on 15 June 2025. During and after data collection, the authors did not have access to information that could identify individual participants; all analyses were performed on anonymized data.

### Inclusion criteria

(1) patients with differentiated thyroid cancer after surgery;
(2) patients received ^131^I therapy for at least 6 months;
(3) patients are willing to receive Tg testing and ^68^Ga-FAPI-04 PET/CT scans. The clinical indications for performing ^68^Ga-FAPI-04 PET/CT were categorized as follows: (a) rising or persistently elevated serum Tg levels despite negative conventional imaging; (b) suspicious findings on anatomical imaging requiring further characterization; (c) routine restaging based on the referring physician’s clinical judgment; and (d) routine follow-up or other reasons.
(4) anti-thyroglobulin autoantibody (TgAb) was negative (less than 15 IU/mL);
(5) patients who have signed an informed consent form (signed by the participant, parent, or legal representative) according to the guidelines of the Clinical Research Ethics Committee.

### Exclusion criteria

(1) patients with history of other malignant tumors;
(2) patients with pregnancy.

### Tracer synthesis

DOTA-FAPI-04 was purchased from MedChemExpress, LLC. ^68^Ga-FAPI-04 was prepared according to a previously described protocol [14]. Radioactive high-performance liquid chromatography demonstrated that the radiochemical purity of ^68^Ga-FAPI-04 was > 98%. The final products, ^68^Ga-FAPI-04, were sterile and pyrogen-free.

### Acquisition of PET/CT images

Patients who underwent ^68^Ga-FAPI-04 PET/CT examinations did not require special preparation. The intravenous doses of ^68^Ga-FAPI-04 were weight-adjusted (1.85 MBq/kg). Patients were required to drink 1,000 mL of water to fill their stomach before examination and to urinate before the PET/CT scan. 60 minutes after intravenous injection [15], PET/CT examination (uMI780, United Imaging Healthcare) was performed from the head (separate head scans for patients with suspected brain metastasis) to the middle thigh. CT scanning was performed with the following parameters: tube voltage, 120 kV; current, 120 mA; layer thickness, 3.00 mm. The scans were reconstructed using a matrix size of 128 × 128 pixels. Data were uploaded to a post-processing workstation (version R002, uWS-MI, United Imaging Healthcare) for processing. All PET images required iterative reconstructions.

### Image analysis of PET/CT

^68^Ga-FAPI-04 PET/CT images were evaluated by two experienced nuclear medicine doctors. Any differences in opinion were resolved through consultation. On PET images, a lesion was considered positive if the focal area of ^68^Ga-FAPI-04 uptake was visually higher than the background. The location, number, and SUV_max_ value of positive lesions were recorded. We evaluated recurrence, lymph node metastasis, and distant metastasis of DTC. Calculating target-to-background ratio (TBR) and the best classifying threshold.

The uptake of ^68^Ga-FAPI-04 in lesions is quantified using the SUV_max_ and the TBR. The TBR of relapse lesions and lymph node metastasis are calculated using the background uptake of the mediastinum, liver, and spleen, respectively, while the metastasis to other tissues uses the corresponding non-tumor tissue background (lung lesions use lung as background, pleural lesions use pleural background, and bone lesions use bone background). The maximum of TBR from all lesions is taken as the patient’s TBR_max_, which is used to calculate the Receiver Operating Characteristic (ROC) curve to determine the best threshold. Patients are diagnosed and grouped based on TBR, and a comparison is made with groups based on Tg values to assess the predictive accuracy of both methods.

### Statistical analysis

We represent the background SUV_max_ of the mediastinum, liver, and spleen as medians and ranges, and we use the Wilcoxon signed rank test to evaluate the differences among them. A two-tailed P < 0.05 is considered statistically significant. Additionally, a 10-fold cross-validation was performed to internally validate the derived TBR threshold.

### Reference standards

All ^68^Ga-FAPI-04 PET/CT findings were compared with the reference standards. Histopathological biopsy results served as the definitive reference standard for final diagnoses. Biopsies were conducted on suspicious lesions in 29 participants. For those patients who did not undergo biopsy, alternative reference standards included serial serum Tg monitoring over a minimum period of one year, clinical examination outcomes, and conventional imaging modalities such as ultrasound, chest CT, and MRI, to corroborate the PET/CT findings. For the 118 patients who did not undergo biopsy, the median follow-up duration was 18.30 months (interquartile range: 14.20–24.10 months). The criteria for positive disease according to Fu et al [13] [2] with minor modifications as follows: (1) participants with stable Tg levels (Tg_off_＞1 ng/mL or Tg_on_＞10 ng/mL); (2) participants with a serial rise in Tg levels without any intervention; and (3) clinical examination and conventional imaging suggestive of the presence of metastatic disease. The criteria for negative disease were as follows: (1) spontaneous decrease in Tg levels without any intervention and a low value at follow-up and (2) negative clinical examination and conventional imaging findings for the presence of metastatic disease. PET/CT findings were classified as true positive (TP), true negative (TN), false positive (FP), or false negative (FN) based on the reference standards.

## Results

### Patient characteristics

The final study sample included 147 participants, with a median age of 50 years, ranging from 17 to 76 years, consisting of 50 men (34%) and 97 women (66%). The enrolled patients included 145 papillary carcinomas and 2 follicular carcinoma. The participant characteristics are summarized in Table 1. The study included 48 patients with stimulated TSH (TSH_on_) and 99 patients with repressed TSH (TSH_off_), with a median TSH_on_ level of 38.86 mIU/L (range: 30.15–100.00 mIU/L) and a median TSH_off_ level of 0.03 mIU/L (range: 0.01–0.07 mIU/L). Additionally, the median Tg_on_ level is 2.8 ng/mL (range: 0.04–500.00 ng/mL) and the median Tg_off_ level is 0.12 ng/mL (range: 0.04–480.00 ng/mL). Based on medical record review, the distribution of clinical indications for ^68^Ga-FAPI-04 PET/CT was: Tg elevation with negative imaging (10 patients, 6.80%); suspicious imaging findings (32 patients, 21.77%); physician-judged high-risk restaging (70 patients, 47.62%); and routine follow-up or other reasons (35 patients, 23.81%). Thus, the cohort had a high pre-test probability, with an overall positive scan rate of 38.78% (57/147). Among these patients, 29 were diagnosed with recurrent or metastatic lesions through biopsy, while 118 were diagnosed via follow-up assessments. Uptake of ^68^Ga-FAPI-04 was observed to be positive in 57 patients, of whom 46 were Tg-positive and 11 were Tg-negative. Conversely, ^68^Ga-FAPI-04 uptake was negative in 90 patients, all of whom were Tg-negative.

**Table 1:** Participant characteristics.

Characteristic	
**Median age (years)**	50 (17-76)
＜55	33.87 ± 6.74
≥ 55	59 (55-76)
**Sex(%)**	
M	50 (34.0)
F	97 (66.0)
**Pathologic subtype (%)**	
Papillary	145 (98.6)
Follicular	2 (1.4)
**TSH**_**on**_ **(IU/mL)**	48/38.86 (30.15-100.00)
**Tg**_**on**_ **(IU/mL)**	0.12 (0.04-480.00)
**TSH**_**off**_ **(IU/mL)**	99/2.80 (0.04-500.00)
**Tg**_**off**_ **(IU/mL)**	0.03 (0.01-0.07)
**The** ^**68**^**Ga-FAPI-04 PET/CT scanning result (%)**	
FAPI-positive (%)	57 (38.8)
Tg-positive	46 (80.7)
Tg-negative	11 (19.3)
FAPI-negative (%)	90 (61.2)
Tg-positive	0 (0.0)
Tg-negative	90 (100.0)
**Method of confirmation (%)**	
Histopathologic results	29 (19.7)
Follow-up results	118 (80.3)

Demographic and clinical characteristics of the study participants. Categorical variables are presented as count (and percentage). Quantitative data are shown as mean ± standard deviation or median (min to max), depending on the normality of their distribution. Tg = thyroglobulin, TSH_on_= TSH-stimulated, TSH_off_=TSH-repressed, Tg_off_=thyrotropin-stimulated thyroglobulin, Tg_on_= thyrotropin suppressed thyroglobulin.

### Overall diagnostic performance

**Fig 1:**
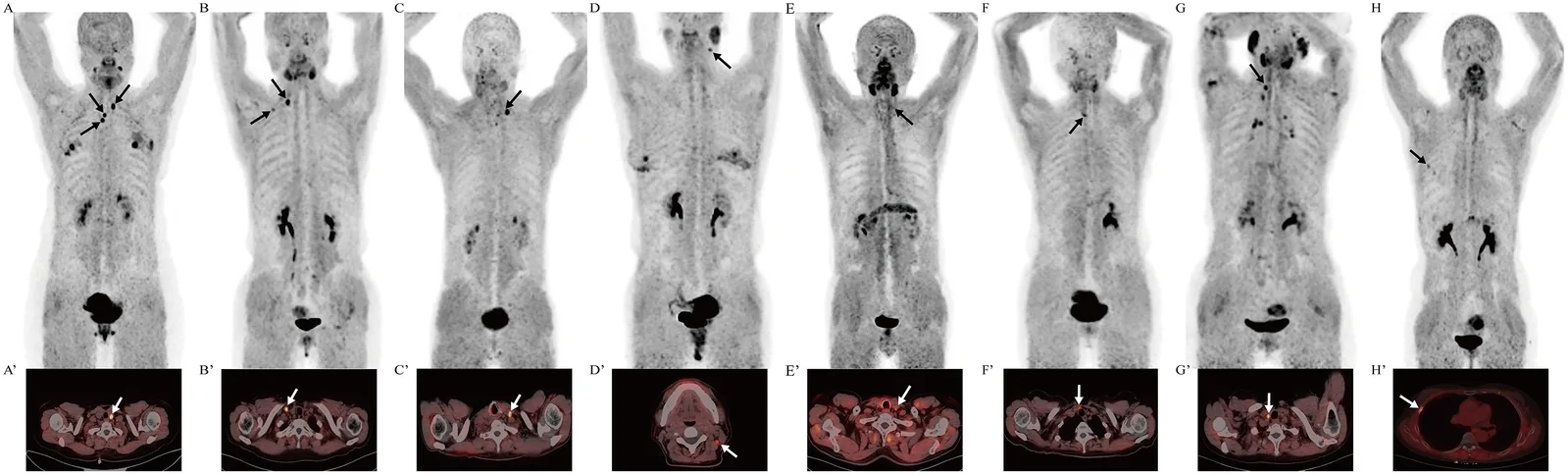
Positive ⁶⁸Ga-FAPI-04 PET/CT Findings in Tg-Negative DTC Patients. (A-H) Representative true-positive ⁶⁸Ga-FAPI-04 PET/CT findings in eight DTC patients with false-negative Tg levels. (A-F) PET/CT MIP showing lymph nodes (arrows) in six patients, all subsequently confirmed as metastatic by fine-needle aspiration pathology. (G) MIP image showing a lymph node in right Level VII and scattered metastatic nodules in both lungs. (H) MIP image showing an osteolytic lesion in the right 4th axillary rib. (A’-H’) Corresponding transverse PET/CT fusion images provide precise anatomical localization of the lesions. Detailed findings are as follows: (A) A 33-year-old female, 6 months post-operation (Tg_off_, 0.15 ng/mL), with nodes in left Level IV (SUV_max_, 12.13), Level VI (SUV_max_, 8.97), and mediastinal station 3A (SUV_max_: 9.40). (A’) Transverse PET/CT fusion image of the node in left Level IV. (B) A 58-year-old female, 11 months post-operation (Tg_off_, 0.04 ng/mL), with nodes in right Level VB (SUV_max_, 7.39) and Level VII (SUV_max_, 2.79). (B’) Transverse PET/CT fusion image of the node in right Level VB. (C) A 52-year-old male, 7 months post-operation (Tg_on_, 0.8 ng/mL), with a node in left Level IV (SUV_max_, 8.16). (C’) Transverse PET/CT fusion image of the node in left Level IV. (D) A 48-year-old female, 6 months post-operation (Tg_off_, 0.14 ng/mL), with a node in left Level II (SUV_max_, 5.6). (D’) Transverse PET/CT fusion image of the node in left Level II. (E) A 57-year-old male, 15 months post-operation (Tg_off_, 0.15 ng/mL), with a node in left Level VB (SUV_max_, 2.62). (F) A 60-year-old female, 11 months post-operation (Tg_off_, 0.08 ng/mL), with a node in right Level VI (SUV_max_, 3.41). (E’) Transverse PET/CT fusion image of the node in left Level VB. (G) A 76-year-old female, 19 months post-operation (Tg_off_, 0.04 ng/mL), showing a node in right Level VII (arrow, SUV_max_, 5.45) and scattered metastatic nodules in both lungs (arrow, SUV_max_, 1.24). Subsequent follow-up showed persistently elevated Tg and imaging findings consistent with metastasis. (G’) Transverse PET/CT fusion image of the node in right Level VII. (H) A 52-year-old female, 10 months post-operation (Tg_off_, 0.04 ng/mL), showing an osteolytic lesion in the right 4th axillary rib with elevated uptake (arrow, SUV_max_, 2.56), later confirmed as bone metastasis by pathology. (H’) Transverse PET/CT fusion image of the osteolytic lesion in the right 4th axillary rib.

We evaluated the diagnostic efficacy of ^68^Ga-FAPI-04 PET/CT and Tg under conditions of both suppressed and stimulated TSH, as detailed in Table 2. In the TSH_off_ state, the sensitivity, specificity, positive predictive value, negative predictive value, and accuracy of ^68^Ga-FAPI-04 PET/CT were 97.14%, 95.31%, 91.89%, 98.39%, and 95.96%, respectively. The corresponding metrics for Tg were 77.14%, 100%, 100%, 88.89%, and 91.92%. Under TSH_on_ conditions, ^68^Ga-FAPI-04 PET/CT demonstrated a sensitivity, specificity, positive predictive value, negative predictive value, and accuracy of 100%. For Tg, the sensitivity, negative predictive value, and accuracy were 95%, 96.55%, and 97.92%, respectively, with both specificity and positive predictive value were 100%. Among the true-positive cases identified by ^68^Ga-FAPI-04 PET/CT, 8 patients exhibited metastatic lesions despite having negative serum Tg levels. Specifically, six cases involved lymph node metastases confirmed via pathological examination (Fig 1A–F), one case involved both lymph node and lung metastases corroborated by imaging follow-up and a subsequent increase in Tg levels (Fig 1G), and one case involved a bone metastasis confirmed by pathology (Fig 1H). These observations suggest that ^68^Ga-FAPI-04 PET/CT may offer superior diagnostic performance compared to Tg monitoring in the detection of metastases in differentiated thyroid cancer.

**Table 2:** Lesion-based Diagnostic Accuracy of ^68^Ga-FAPI-04 PET/CT and Tg.

	^68^Ga-FAPI-04 PET/CT		Tg	
	TSH_on_	TSH_off_	TSH_on_	TSH_off_
**Sensitivity**	100%	97.14%	95.00%	77.14%
**Specificity**	100%	95.31%	100%	100%
**Positive Predictive Value**	100%	91.89%	100%	100%
**Negative Predictive Value**	100%	98.39%	96.55%	88.89%
**Accuracy**	100%	95.96%	97.92%	91.92%

Lesion-based diagnostic performance of ^68^Ga-FAPI-04 PET/CT and serum Tg under different TSH Conditions. PET/CT = positron emission tomography computed tomography, Tg = thyroglobulin, TSH_on_= TSH-stimulated, TSH_off_ = TSH-repressed.

**Fig 2:**
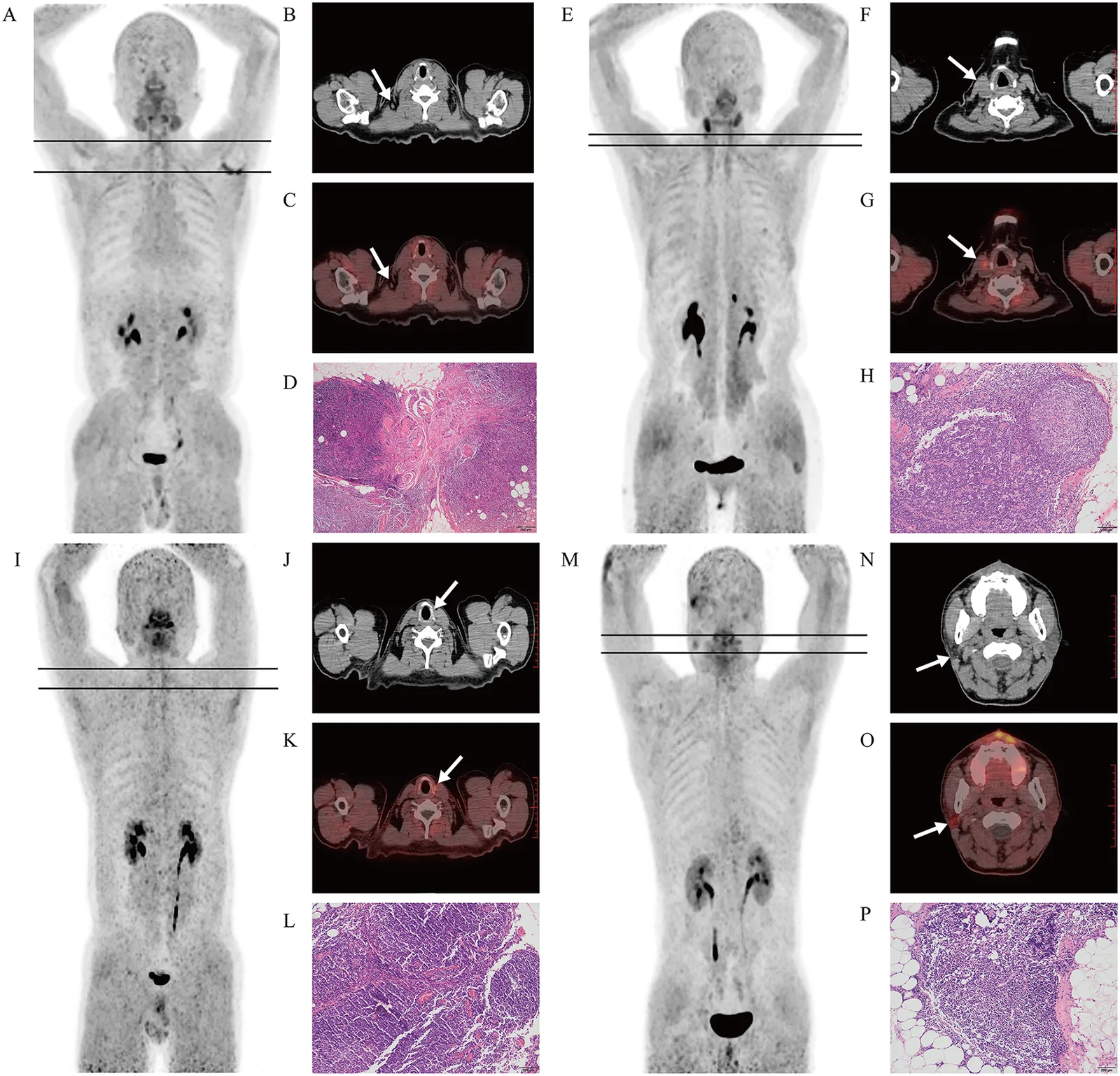
False-Negative and False-Positive ^68^Ga-FAPI-04 PET/CT Findings. (A-D) A case of false-negative result. (A) MIP image. (B-C): Axial PET/CT and PET images show a lymph node in right Level IV (thick arrow) without significant radiotracer uptake (SUV_max_, 1.46). (D) Cytology from fine-needle aspiration confirmed metastatic papillary carcinoma (hematoxylin and eosin stain). (E-P) Three representative cases with false-positive (inflammatory) findings. (E) MIP image. (F-G) Axial PET/CT and PET images demonstrate focal ⁶⁸Ga-FAPI-04 uptake (thick arrow) in right Level VI (SUV_max_, 3.57). (H) Histopathology revealed inflammatory changes. (I) MIP image. (J-K) Axial images show increased uptake (thick arrow) in left Level VI (SUV_max_, 2.70). (L) Pathology confirmed inflammatory changes. (M) MIP image. (N-O) Axial images show focal uptake (thick arrow) in right Level II (SUV_max_, 2.29). (P) Pathology indicated inflammatory changes.

To address potential incorporation bias, we performed a subgroup analysis restricted to the 29 patients with histopathological confirmation. In the TSH_on_ subgroup (n = 9), there were no reference‑standard negative patients; ^68^Ga‑FAPI‑04 PET/CT showed 100% sensitivity (9/9), 100% PPV (9/9), and 100% accuracy, while serum Tg showed 88.89% sensitivity (8/9), 100% PPV (8/8), 0% NPV (0/1), and 88.89% accuracy. In the TSH_off_ subgroup (n = 20), ^68^Ga‑FAPI‑04 PET/CT demonstrated 94.12% sensitivity (16/17), 84.21% PPV (16/19), 0.00% specificity (0/3), 0% NPV (0/1), and 80.00% accuracy. The three false‑positive findings were inflammatory lymph nodes (Fig 2G,2K,2O) and the single false‑negative was a small lymph node (Fig 2D). Serum Tg showed 52.94% sensitivity (9/17), 100% specificity (3/3), 100% PPV (9/9), 27.27% NPV (3/11), and 60.00% accuracy. Despite the extreme specificity and NPV values in the TSH_off_ subgroup, these reflect the very small numbers of true‑negative (n = 3) and FAPI‑negative (n = 1) cases, and the fact that all false‑positive and false‑negative cases of the entire study were concentrated in this small subgroup. The full‑cohort analysis (Table 2) provides more stable estimates, with FAPI specificity of 95.31% and NPV of 98.39% for the TSH_off_ state.

### Lesion-based diagnostic accuracy of ^68^Ga-FAPI-04 PET/CT

As demonstrated in Table 3, among the 57 patients exhibiting positive findings on ^68^Ga-FAPI-04 PET/CT scans, the following lesions were identified and subsequently validated: (1) Thyroid recurrence was detected in 5 patients, with a mean SUV_max_ of 10.19 ± 5.55; these diagnoses were confirmed through either pathological examination or follow-up. (2) A total of 101 positive lymph nodes were identified in 41 patients, with a median SUV_max_ of 4.4 (range: 1.50–15.30). Among these, 38 nodes were confirmed as metastatic via pathology or follow-up, while 3 were determined to be false positives. As depicted in Fig 2, these positive lymph nodes were located adjacent to the right Level VI (SUV_max_: 3.57, Fig 2G), within the left Level VI (SUV_max_: 2.70, Fig 2K), and in the right Level II (SUV_max_: 2.29, Fig 2O); fine-needle aspiration pathology indicated inflammatory changes. (3) A total of 86 positive nodules were identified in 15 patients, with a median SUV_max_ of 1.67 (range: 0.75–7.48), and follow-up results were consistent with initial findings. (4) Thirteen bone-positive lesions were detected in 6 patients, with a mean SUV_max_ of 2.46 ± 0.84, and follow-up results corroborated these findings. (5) Lastly, one patient presented with pleural metastasis, characterized by an SUV_max_ of 4.5, with the diagnosis confirmed upon follow-up. Additionally, one false-negative case was observed in this study. Two months following ^131^I therapy, a neck ultrasound revealed an abnormal lymph node measuring approximately 0.7 cm in diameter located in the right cervical level IV. Fine-needle aspiration subsequently confirmed the presence of metastatic papillary carcinoma. However, no appreciable uptake was seen on ^68^Ga-FAPI-04 PET/CT (Fig 2A).

**Table 3:** Sites of lesion detection, radiotracer avidity and concordance/ discordance on ^68^Ga-FAPI-04 PET/CT.

Site	Lesion number (Number of patients)	SUV_max_	Concordant lesions (Number of patients)	Discordant lesions(Number of patients)
**Recurrence of thyroid**	5 (5)	10.19 ± 5.55	5	0
**Lymph node metastases**	101 (41)	4.40 (1.50-15.30)	38	3
**Lung metastases**	86 (15)	1.67 (0.75-7.48)	15	0
**Bone metastases**	13 (6)	2.46 ± 0.84	6	0
**Pleura metastases**	1	4.5	1	0

Anatomical distribution, radiotracer Avidity, and concordance of lesions detected by ^68^Ga-FAPI-04 PET/CT. Data are shown as the number of cases, mean ± standard deviation or median (min to max), depending on the normality of their distribution. SUV_max_ = maximum standardized uptake value, PET/CT = positron emission tomography computed tomography.

### Analysis of the diagnostic efficacy of lymph node TBR under different organ backgrounds

**Fig 3:**
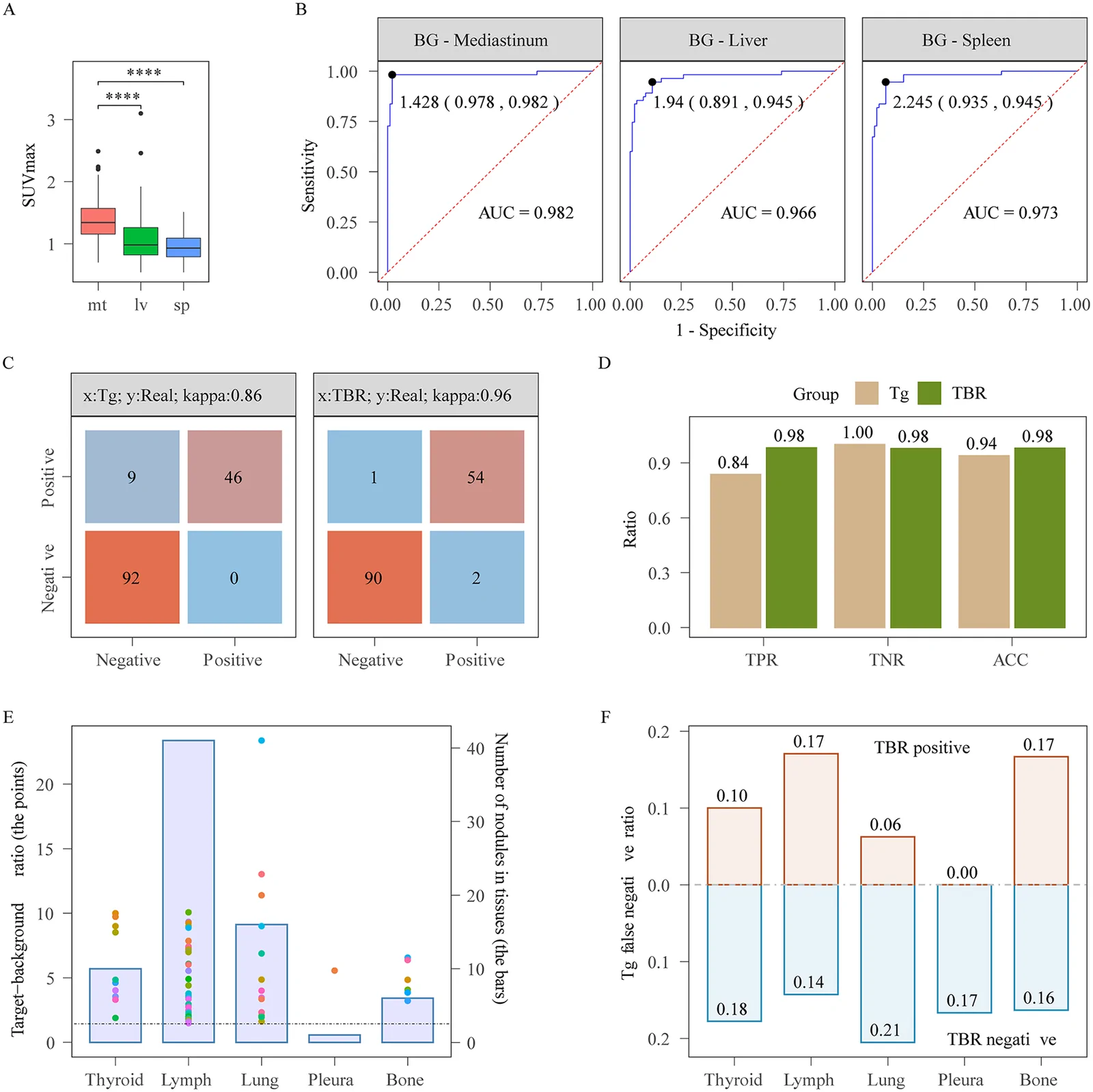
Diagnostic Performance of TBR Compared to Tg. (A) The background SUV_max_ of mediastinum, liver, and spleen. P-values were calculated using Wilcoxon signed rank test. ****: p < 0.0001. (B) Receiver Operating Characteristic (ROC) curve for the TBR-based classifying. The TBR of lymph node metastasis are calculated using the background uptake of the mediastinum, liver, and spleen, respectively, while the metastasis to other tissues uses the corresponding non-tumor tissue background. The AUC and threshold are noted. (C) Kappa values for diagnostic grouping based on Tg and TBR. The y-axis represents the true diagnostic results, while the x-axis represents the Tg diagnostic results (left panel) and the TBR diagnostic results (right panel). (D) True positive rate (TPR), true negative rate (TNR), and accuracy (ACC) based on Tg and TBR diagnostic results. (E) Distribution of lesions based on TBR diagnostic results. (F) The distribution of lesions in 9 cases of false negatives from the Tg diagnostic results (Fig 3C). The orange bars on top represent the proportion of Tg false negative patients among TBR positive patients, while the blue bars below represent the proportion of Tg false negative patients among TBR negative patients. The x-axis indicates whether there is metastasis to the tissue or relapse in the patients.

Given that imaging-based diagnosis of lymph node metastasis is susceptible to false positive or false negative results, we evaluated the optimal diagnostic threshold of the lymph node TBR using the mediastinum, liver, and spleen as backgrounds, in order to explore feasible pathways for improving the diagnostic accuracy of ^68^Ga-FAPI-04 PET/CT. We collected a total of 147 cases of DTC patients, where the SUV_max_ of the mediastinum as background uptake was significantly higher than that of the other two tissues (liver and spleen) (Fig 3A). We calculated the TBR of relapse and lymph node metastases using the mediastinum, liver, and spleen as backgrounds respectively, and together with the TBR values of other lesions, we took the maximum TBR for each patient to calculate the ROC curve. The results showed that the diagnostic effect was best when using the mediastinum as background for calculating the TBR of relapse and lymph node metastases, with the optimal threshold being 1.428 (Fig 3B). To assess the potential overfitting of this threshold, a 10-fold cross-validation was performed. The mean cross-validated AUC was 0.974 (95% CI: 0.944–1.000), with a mean sensitivity of 0.980 and mean specificity of 0.972, confirming the robustness of the threshold (S1 Fig). Comparing the diagnostic results based on TBR (with the mediastinum as the background for relapse and lymph node metastases) with those based on Tg, we found that the kappa value of the TBR-based diagnosis (0.96) was higher than that of the Tg diagnosis (0.86) (Fig 3C). The accuracy of the TBR-based diagnosis was 0.98, while the Tg-based accuracy was 0.94, with the true positive rate (TPR) of the Tg-based diagnosis (0.84) being significantly lower than that of the TBR-based diagnosis (0.98) (Fig 3D). Using TBR as the diagnostic criterion, we analyzed the distribution of relapse and metastasis in patients and found that the main type of metastasis in DTC patients was lymphatic, followed by pulmonary metastasis, while the relapse rate, bone metastasis, and pleural metastasis were relatively low (Fig 3E). By analyzing whether there were relapses or metastases for patients with false negatives in Tg diagnosis, we found that patients with lymph node metastasis were more likely to show Tg false negatives, whereas patients with pulmonary metastasis were less likely to present with Tg false negatives (Fig 3F). Our results indicate that using TBR with the mediastinum as the background for relapse and lymph node metastasis can effectively diagnose patients’ relapse and metastasis, outperforming Tg-based diagnostic standards.

## Discussion

^68^Ga-FAPI-04 emerges as a promising imaging agent for tumor diagnosis, facilitating the visualization of the stromal components within the tumor microenvironment. Its application in the diagnosis of thyroid cancer has been documented in the literature, with a particular emphasis on iodine-refractory thyroid cancer. Nevertheless, the prospective utilization of ^68^Ga-FAPI-04 as a standard diagnostic tool for DTC patients following ^131^I treatment has yet to be thoroughly investigated. To address this gap, we conducted a randomized study involving 147 patients who underwent ^68^Ga-FAPI-04 PET/CT imaging to evaluate its value in the follow-up of DTC patients. Our findings indicate that ^68^Ga-FAPI-04 demonstrates high diagnostic efficacy for detecting disease recurrence and metastasis in both Tg-positive and Tg-negative patients.

Based on American Thyroid Association guideline recommendations, ^18^F-FDG PET/CT should be considered when an empirical dose of ^131^I fails to localize the disease [16]. ^68^Ga-FAPI-04 PET/CT, which targets fibroblast activation protein expressed in the tumor stroma, has been reported to demonstrate high diagnostic efficiency in various malignancies. Given its different biological target, ^68^Ga-FAPI-04 PET/CT may serve as a complementary tool to ^18^F-FDG PET/CT in the evaluation of DTC patients, particularly when FDG results are equivocal or negative despite clinical suspicion [17]. Additionally, post-treatment ^131^I imaging exhibits low resolution and limited diagnostic efficacy for detecting smaller lesions, numerous patients did not show any positive lesions on ^131^I imaging. Therefore, we did not compare post-treatment ^131^I imaging with ^68^Ga-FAPI-04 PET/CT.

Patients with DTC often need to temporarily discontinue Euthyrox in order to accurately assess their serum Tg levels, which will lead to the development of hypothyroid symptoms, adversely affecting patient adherence to the treatment regimen. Consequently, a significant number of patients may be reluctant to discontinue Euthyrox. In this study, 67% of the patients enrolled were in a state of Tg suppression, the ^68^Ga-FAPI-04 PET/CT demonstrated superior diagnostic efficiency compared to Tg, achieving an accuracy of 95.96% versus 91.92% of Tg. This study involved 48 patients with TSH_on_ status, comprising 29 Tg-negative and 19 Tg-positive patients, the diagnostic accuracy for Tg was 97.92%, whereas ^68^Ga-FAPI-04 PET/CT achieved a 100% diagnostic accuracy for all patients. These findings indicate that ^68^Ga-FAPI-04 PET/CT maintains high diagnostic efficacy in patients with DTC, irrespective of levothyroxine discontinuation, thereby positioning it as a promising imaging modality (Table 2).

Furthermore, we found that in eight patients with negative Tg levels, ^68^Ga-FAPI-04 PET/CT was more effective in detecting metastatic lesions (Fig 1). Traditionally, Tg levels have been regarded as a crucial marker for assessing the risk of recurrence in thyroid cancer patients. However, Tg levels may not always accurately reflect the presence or progression of tumors. Previous research has indicated that radiolabeled iodine imaging-positive lesions can exist even when Tg levels are low or undetectable [18]. Additionally, the presence of heterophile antibodies can result in false negative or false positive Tg measurements [19]. These findings suggest that relying solely on Tg levels may be inadequate for a comprehensive evaluation of disease. As an innovative imaging modality, ^68^Ga-FAPI-04 PET/CT offers the potential for earlier detection of metastatic lesions, thereby enhancing the overall assessment of DTC patients.

A limited number of lymph nodes demonstrate false positives in ^68^Ga-FAPI-04 imaging in our study (Fig 2B-D), a challenge that arises due to the elevated energy consumption associated with the functional activity of inflammatory cells [14]. Furthermore, we identified an instance in which a lymph node exhibited a false-negative result on the ^68^Ga-FAPI-04 PET/CT, potentially attributable to the small short-axis diameter of lymph node. This indicates that exclusive reliance on the ^68^Ga-FAPI-04 PET/CT is inadequate for the precise characterization of lesions. Indeed, in clinical practice, no singular indicator can serve as an unequivocal diagnostic criterion. A thorough assessment necessitates the integration of the medical history, imaging studies, laboratory analyses, and pathological evaluations. It is imperative that all indicators consider potential false-negative and false-positive factors to prevent misdiagnosis and unwarranted treatment. To mitigate the risk of over‑interpretation, we emphasize that ^68^Ga‑FAPI‑04 PET/CT findings should be correlated with conventional imaging, such as ultrasonography, and when clinically warranted, verified by cytology or histopathology before surgical decision‑making. Nonetheless, the diagnostic accuracy of ^68^Ga-FAPI-04 PET/CT in assessing lymph node metastasis remains substantial. This conclusion aligns with the findings of Fu et al [20], who reported that ^68^Ga-FAPI-04 PET/CT exhibits high diagnostic efficacy for detecting recurrence and metastasis in differentiated thyroid cancer, particularly lymph node metastasis.

Therefore, determining the lymph node lesion TBR threshold is crucial for differentiating between benign and malignant lesions, and also aids in assessing treatment responses. The TBR threshold value may vary depending on the type of cancer. For instance, in the prediction of neoadjuvant chemotherapy for breast cancer, research has found that the TBR of ^68^Ga-FAPI-04 PET/CT has predictive value at different stages. The optimal preoperative TBR critical value is 1.4 (AUC = 0.971), while the optimal post-chemotherapy TBR critical value is 7.6 (AUC = 0.848), which can be used to evaluate the likelihood of pathological complete response [21]. Additionally, the selection of the organ used as the background reference is a critical factor. In this study, we selected three commonly used background tissues, including the average SUV_max_ values of the mediastinum, liver, and spleen. We then compared the TBR values of the Lymph node lesions, against these three different backgrounds using ROC curve analysis. Ultimately, we found that the TBR value using the mediastinum as the background had a higher sensitivity and positive predictive value, and when the TBR threshold value was greater than 1.428 (AUC = 0.982), providing the best diagnostic threshold (Fig 3).

This study is subject to several limitations. Firstly, the number of patients included in this study was small, and not all lesions were confirmed by biopsy. However, a subgroup analysis of the 29 biopsy‑proven patients (S1 Table) showed that the sensitivity of ^68^Ga‑FAPI‑04 PET/CT remained high, consistent with the full‑cohort analysis (Table 2). The extreme specificity and NPV observed in the TSH_off_ subgroup were due to the very limited number of true‑negative and FAPI‑negative cases in this small sample. In routine clinical practice, biopsy is performed only when imaging or Tg levels raise suspicion. Thus, a biopsy‑proven cohort is inherently enriched for true‑positive and false‑positive cases, while true‑negative cases are rare. Second, the relatively high positive scan rate (38.78%) indicates a higher pre‑test probability than a typical unselected DTC follow‑up population. This selection bias may overestimate PPV and underestimate NPV. Therefore, prospective studies in consecutive unselected patients are warranted. Third, this study did not include a head‑to‑head comparison with ^18^F‑FDG PET/CT, although previous studies have suggested potential advantages of ^68^Ga-FAPI-04 over ^18^F-FDG PET/CT in DTC, the absence of direct comparative data in our cohort limits any claims of superiority. Future prospective studies with paired ^18^F‑FDG and ^68^Ga-FAPI-04 imaging are needed to determine the optimal imaging strategy. Fourth, the optimal TBR threshold of 1.428 was derived and internally validated within this single-center cohort. However, external validation in an independent, preferably multicenter cohort is necessary to confirm the generalizability of our findings.

## Conclusions

Regardless of Tg status, ^68^Ga-FAPI-04 PET/CT demonstrated high diagnostic efficacy for detecting local recurrences and lymph node, bone, pleural, and pulmonary metastases in DTC patients after ^131^I therapy. This imaging modality can serve as a significant complement to routine Tg monitoring.

## Supporting information

S1 Fig Ten-fold cross-validation ROC curves for the TBR-based diagnostic model. The mean AUC was 0.974 (95% CI: 0.944–1.000), confirming internal validity of the TBR threshold with minimal overfitting.
(TIF)

S1 Table Lesion‑based diagnostic accuracy of ^68^Ga‑FAPI‑04 PET/CT in 29 histopathologically confirmed DTC patients. Lesion-based diagnostic performance of ^68^Ga-FAPI-04 PET/CT and serum Tg in the biopsy-confirmed subgroup of 29 DTC patients. In the TSH_off_ subgroup (n = 20), all three false‑positive FAPI findings corresponded to inflammatory lymph nodes (Fig 2G, 2K, 2O) and the single false‑negative finding to a lymph node (Fig 2D). The 0.00% specificity and 0.00% NPV reflect the very small numbers of true‑negative (n = 3) and FAPI‑negative (n = 1) cases and do not represent true diagnostic performance (see Table 2 for comparison). N/A^*^: not applicable due to absence of reference-standard negative patients in the TSH_on_ subgroup.
(DOCX)
