# Supplementary material for: 68Ga-FAPI-04 PET/CT has high diagnostic efficacy in Tg-positive and Tg-negative differentiated thyroid cancer
Source: PLoS One. 2026 Sep 3;21(9):e0355343. doi: 10.1371/journal.pone.0355343 (PMC13541195; doi:10.1371/journal.pone.0355343)
Supplement: Supplementary file 1 [file pone.0355343.s002.docx]

| **S1 Table. Lesion‑based diagnostic accuracy of ^68^Ga‑FAPI‑04 PET/CT in 29 histopathologically confirmed DTC patients.** | | | | |
| --- | --- | --- | --- | --- |
|  | **^68^Ga-FAPI-04 PET/CT** | | **Tg** | |
|  | TSH_on_ | TSH_off_ | TSH_on_ | TSH_off_ |
| **Sensitivity** | 100% | 94.12% | 88.89% | 52.94% |
| **Specificity** | N/A^*^ | 0.00% | N/A^*^ | 100% |
| **Positive Predictive Value** | 100% | 84.21% | 100% | 100% |
| **Negative Predictive Value** | N/A^*^ | 0.00% | 0.00% | 27.27% |
| **Accuracy** | 100% | 80.00% | 88.89% | 60.00% |
| Lesion-based diagnostic performance of ^68^Ga-FAPI-04 PET/CT and serum Tg in the biopsy-confirmed subgroup of 29 DTC patients. In the TSH_off_ subgroup (n=20), all three false‑positive FAPI findings corresponded to inflammatory lymph nodes (Figure 2G, 2K, 2O) and the single false‑negative finding to a lymph node (Figure 2D). The 0.00% specificity and 0.00% NPV reflect the very small numbers of true‑negative (n=3) and FAPI‑negative (n=1) cases and do not represent true diagnostic performance (see Table 2 for comparison). N/A^*^: not applicable due to absence of reference-standard negative patients in the TSH_on_ subgroup. | | | | |
