# Supplementary material for: 68Ga-FAPI-04 PET/CT has high diagnostic efficacy in Tg-positive and Tg-negative differentiated thyroid cancer
Source: PLoS One. 2026 Sep 3;21(9):e0355343. doi: 10.1371/journal.pone.0355343 (PMC13541195; doi:10.1371/journal.pone.0355343)
Supplement: Supplementary file 2 [file pone.0355343.s003.docx]

Dear Academic Editor Dr. Francesco Dondi and Reviewers,

Thank you for your thoughtful comments and valuable suggestions on our manuscript (PONE-D-26-18193). We have carefully addressed all points raised by the editor and reviewers. Below we provide point-by-point responses. All changes in the revised manuscript are marked using track changes.

Response to Academic Editor

1. Please ensure that your manuscript meets PLOS ONE's style requirements, including those for file naming.

**Reply:** We have revised the manuscript to meet PLOS ONE’s formatting guidelines.

2. PLOS requires an ORCID iD for the corresponding author in Editorial Manager on papers submitted after December 6th, 2016. Please ensure that you have an ORCID iD and that it is validated in Editorial Manager. To do this, go to ‘Update my Information’ (in the upper left-hand corner of the main menu), and click on the Fetch/Validate link next to the ORCID field. This will take you to the ORCID site and allow you to create a new iD or authenticate a pre-existing iD in Editorial Manager.

**Reply:** The corresponding author’s ORCID iD has been verified in Editorial Manager.

3. Thank you for stating the following financial disclosure:“This work was supported by the Youth Fund of Southwest Medical University (Grant No. 2021ZKQN054 and Grant No. 2022QN055 ).” Please state what role the funders took in the study. If the funders had no role, please state: "The funders had no role in study design, data collection and analysis, decision to publish, or preparation of the manuscript." If this statement is not correct you must amend it as needed. Please include this amended Role of Funder statement in your cover letter; we will change the online submission form on your behalf.

**Reply:** We have added the following statement in the cover letter: “This work was supported by the Youth Fund of Southwest Medical University (Grant No. 2021ZKQN054 and Grant No. 2022QN055). The funders had no role in study design, data collection and analysis, decision to publish, or preparation of the manuscript. The funding was received by the first author (Lixian Mou, Grant No. 2021ZKQN054) and the corresponding author (Wei Wang, Grant No. 2022QN055), who contributed to study design, data analysis, and manuscript writing as co‑authors.”

4. In this instance it seems there may be acceptable restrictions in place that prevent the public sharing of your minimal data. However, in line with our goal of ensuring long-term data availability to all interested researchers, PLOS’ Data Policy states that authors cannot be the sole named individuals responsible for ensuring data access (<http://journals.plos.org/plosone/s/data-availability#loc-acceptable-data-sharing-methods>). Data requests to a non-author institutional point of contact, such as a data access or ethics committee, helps guarantee long term stability and availability of data. Providing interested researchers with a durable point of contact ensures data will be accessible even if an author changes email addresses, institutions, or becomes unavailable to answer requests. Before we proceed with your manuscript, please also provide non-author contact information (phone/email/hyperlink) for a data access committee, ethics committee, or other institutional body to which data requests may be sent. If no institutional body is available to respond to requests for your minimal data, please consider if there any institutional representatives who did not collaborate in the study, and are not listed as authors on the manuscript, who would be able to hold the data and respond to external requests for data access? If so, please provide their contact information (i.e., email address). Please also provide details on how you will ensure persistent or long-term data storage and availability.

**Reply:** Due to ethical restrictions (participant consent and institutional review board approval), data cannot be publicly shared. Requests for access to the minimal anonymized data set may be directed to the Institutional Data Access / Ethics Committee of the Affiliated Hospital of Southwest Medical University (Contact: Shumao Zhang, Email: 540950842@qq.com, Phone: 0830-3165721). Data will be stored on the hospital's secure server for at least 10 years.

5. If the reviewer comments include a recommendation to cite specific previously published works, please review and evaluate these publications to determine whether they are relevant and should be cited. There is no requirement to cite these works unless the editor has indicated otherwise.

**Reply:** No specific works were recommended for citation. We have uploaded the Response to Reviewers, the revised manuscript with track changes, and a clean manuscript.

Response to Reviewer #1

We thank Reviewer #1 for the positive evaluation and constructive comments on our manuscript. We have carefully addressed all the concerns raised. All changes are marked with track changes in the revised manuscript.

**Comment 1:** Retrospective Design and Potential Bias: As a retrospective study, there is inherent selection bias. Furthermore, only 29 of the 147 participants had their PET/CT findings confirmed via histopathology; the remainder relied on clinical follow‑up, which is less definitive.

**Reply:** We acknowledge these limitations. To address the potential incorporation bias due to limited histopathological confirmation, we performed a subgroup analysis restricted to the 29 biopsy‑proven patients (S1 Table). In this subgroup, the sensitivity of ^68^Ga‑FAPI‑04 PET/CT remained high (94.12-100%), consistent with the full‑cohort analysis. For the 118 clinically followed patients, the median follow‑up duration was 18.3 months (IQR: 14.2-24.1 months), which we believe is sufficient to validate most PET/CT findings. We have explicitly discussed these limitations in the Discussion section.

**Comment 2:** The "Inflammation" Hurdle: Consistent with ^68^Ga‑FAPI’s known limitations, the study reported false positives in lymph nodes due to inflammatory changes. While the authors addressed this through TBR optimization, the risk of 'over‑calling' reactive nodes remains a concern for surgical planning.

**Reply:** We agree with the reviewer that inflammatory false positives remain a challenge for FAPI PET/CT. We have revised the Discussion to explicitly address this limitation. Specifically, we now emphasize that FAPI PET/CT findings should be correlated with conventional imaging (ultrasonography) and, when clinically warranted, verified by cytology or histopathology before surgical decision‑making. This multimodal approach is essential to avoid over‑interpretation and ensure appropriate patient management. All changes are marked in the revised manuscript.

**Comment 3:** Lack of Direct Comparison with ^18^F-FDG: Although the authors cite literature comparing FAPI to FDG, this specific cohort was not tested head‑to‑head. Given that FDG is the current alternative for Tg‑positive/Iodine‑negative (TENIS) cases, a direct comparison would have strengthened the argument for switching to FAPI.

**Reply:** We acknowledge the reviewer’s concern. Only few patients in our cohort underwent ^18^F‑FDG PET/CT within a comparable timeframe, which is an insufficient sample size for a meaningful head‑to‑head analysis. Therefore, we have: (1) softened all claims of superiority over FDG throughout the manuscript; and (2) explicitly added the lack of FDG comparison as a limitation in the Discussion section. We agree that future prospective paired studies are needed to determine the optimal imaging strategy.

**Comment 4:** The authors should make clear in the introduction that ^68^Ga‑FAPI may be useful for TENIS or for any state of DTC.

**Reply:** We agree with the reviewer and have revised the Introduction accordingly. The following sentence has been added (lines 102-104 in manuscript_revised): “Therefore, ^68^Ga‑FAPI‑04 PET/CT may be useful not only in patients with TENIS syndrome but also in various states of DTC, including both Tg‑positive and Tg‑negative conditions, regardless of TSH stimulation status.”

**Comment 5:** Please note that the quality of images in the manuscript is poor, so the reviewer could not see lesions clearly.

**Reply:** We apologize for the poor image quality in the original submission. To comprehensively address this issue, we have taken the following measures:

(1) Added corresponding transverse PET/CT fusion images (A’-H’) in Fig 1 to provide precise anatomical localization of each lesion.
(2) Converted all figures to TIFF format with LZW compression at 600 dpi, following PLOS ONE's specifications, and verified them using the NAAS quality check tool.

Response to Reviewer #2

We thank Reviewer #2 for the thorough and rigorous review.

Major Comments

**Comment 1:** The study reports that only 29 of the 147 patients (19.7%) underwent histopathological biopsy as the definitive reference standard. For the remaining patients, serial serum Tg monitoring over a minimum of one year was utilized alongside imaging to confirm disease status. Because the primary objective of this study is to compare the diagnostic accuracy of ^68^Ga-FAPI-04 PET/CT directly against Tg testing, utilizing Tg as the ground truth to subsequently calculate the sensitivity and specificity of Tg creates a significant incorporation bias. Recommendation: The authors should perform a subgroup analysis restricting the diagnostic performance comparison (FAPI vs. Tg) exclusively to the 29 patients with biopsy-proven results. Furthermore, the median follow-up time for the clinically monitored cohort should be explicitly stated, and this bias must be discussed in the Limitations section.

**Reply:** We thank the reviewer for this important comment. We have performed the requested subgroup analysis on the 29 biopsy‑confirmed patients (S1 Table) and added the median follow‑up time (18.3 months, IQR 14.2-24.1 months) in the Methods and Results sections. We have also expanded the Limitations paragraph to discuss incorporation bias. As shown in S1 Table, the sensitivity of ^68^Ga‑FAPI‑04 PET/CT remained high in both TSH states (100% in TSH_on_, 94.1% in TSH_off_), consistent with the full‑cohort analysis (Table 2). The extreme values (N/A and 0%) in this subgroup are explained by two factors: (i) In clinical practice, biopsy is performed only when recurrence is suspected, so a biopsy‑confirmed cohort naturally lacks true‑negative patients; (ii) All three false‑positive (inflammatory lymph nodes, Figure 2G,2K,2O) and the single false‑negative (a lymph node, Figure 2D) findings of the entire study were concentrated in this small subgroup. Therefore, the 0% specificity and 0% NPV do not indicate poor diagnostic performance of FAPI PET/CT. The full‑cohort analysis (Table 2) demonstrates excellent specificity (95.31% for TSH_off_) and NPV (98.39% for TSH_off_). We have added a detailed discussion of these points in the Results and Discussion sections (see revised manuscript). We believe the incorporation bias does not undermine our main conclusions.

**Comment 2:** The study derives an optimal TBR threshold of 1.428 (AUC = 0.982) for predicting lymph node metastases using the entire patient cohort. Deriving a diagnostic cutoff and reporting its performance metrics on the exact same dataset carries a high risk of statistical overfitting. Recommendation: To strengthen the reliability of this threshold, the authors should perform an internal validation (e.g., K-fold cross-validation or bootstrapping with 1,000 resamples) and report the validated accuracy. If internal validation is not feasible, the lack of an independent validation cohort must be explicitly stated as a major limitation in the Discussion.

**Reply:** We thank the reviewer for this important statistical concern. To address potential overfitting, we performed a 10‑fold cross‑validation. The mean cross‑validated AUC was 0.974 (95% CI: 0.944-1.000; range: 0.836-1.000), with corresponding mean sensitivity of 0.980 and mean specificity of 0.972. The mean AUC was highly consistent with the original value (0.982), confirming that the TBR threshold is robust and not overfitted to our dataset. We have added the cross‑validation methodology to the Statistical Analysis section, the results to the Results section, and a corresponding limitation in the Discussion section. A new S1 Fig showing the cross‑validation ROC curves has also been provided. At the same time, we acknowledge that our findings are derived from a single‑center cohort and lack external validation, which has been explicitly stated as a limitation in the Discussion. Future studies with larger, independent, and preferably multicenter cohorts are warranted to further validate the generalizability of our model.

**Comment 3:** The positive ^68^Ga-FAPI-04 scan rate in this cohort is relatively high at 38.8% (57/147). This suggests that the enrolled population may inherently possess a higher pre-test probability for recurrence or metastasis than a standard post-therapy follow-up population. Recommendation: Please clarify the specific clinical indications for ordering the ^68^Ga-FAPI-04 imaging in the Methods section (e.g., were patients scanned due to suspected recurrence on ultrasound, or as a routine pre-therapy assessment?). The authors should also discuss how this potential selection bias might influence the reported Positive Predictive Value (PPV) and Negative Predictive Value (NPV).

**Reply:** We thank the reviewer for this important observation. We agree that the 38.78% positive scan rate in our cohort indicates a higher pre‑test probability than an unselected DTC follow‑up population. To address this, we have taken the following steps: In the Methods section (Inclusion criteria), we have explicitly defined the clinical indications for performing ^68^Ga‑FAPI‑04 PET/CT, categorizing them into four groups. In the Results section (Patient characteristics), we have reported the distribution of these indications (Tg elevation with negative imaging, suspicious imaging findings, physician‑judged high‑risk restaging, and routine follow‑up or other reasons). In the Discussion section (Limitations), we have added a specific limitation (Second) acknowledging that this selection bias may overestimate PPV and underestimate NPV, and that prospective studies in consecutive unselected patients are warranted.

**Comment 4:** The manuscript frequently references the limitations of ^18^F-FDG PET/CT and cites literature suggesting FAPI's superiority in DTC. However, the current study does not provide direct comparative data. Recommendation: If a subset of the enrolled patients underwent ^18^F-FDG PET/CT within a similar timeframe, incorporating a head-to-head comparative analysis would significantly elevate the manuscript's impact. If such data is entirely unavailable, the authors should soften claims regarding comparative superiority and list the absence of ^18^F-FDG data as a limitation.

**Reply:** We thank the reviewer for this suggestion. Since only few patients had ^18^F-FDG PET/CT within a comparable timeframe (insufficient for analysis), we have:

(1). Softened all claims of superiority over ^18^F-FDG. In the Introduction, “superior sensitivity” was changed to “may have higher sensitivity”. In the Discussion, we removed the detailed criticism of ^18^F-FDG limitations and now state that ^68^Ga-FAPI-04 “may serve as a complementary tool” to ^18^F-FDG.

(2). Added the lack of ^18^F-FDG comparison as a limitation. The revised limitation (now “Third”) reads: “Third, this study did not include a head‑to‑head comparison with ^18^F‑FDG PET/CT, Although previous studies have suggested potential advantages of ^68^Ga-FAPI-04 over ^18^F-FDG PET/CT in DTC, the absence of direct comparative data in our cohort limits any claims of superiority. Future prospective studies with paired ^18^F‑FDG and ^68^Ga-FAPI-04 imaging are needed to determine the optimal imaging strategy”. All changes are marked in the revised manuscript.

**Comment 5:** There are some spelling mistakes and inconsistent formats in the original manuscript.

(1). Table 1: Under the "Pathologic subtype" category, the term "Follicula" is misspelled and should be corrected to "Follicular".

(2). The manuscript uses inconsistent formatting and abbreviations for thyroglobulin states, interchanging Tgoff, Tgofr, and Tgoff. Please rigorously standardize these terms (e.g., consistently using Tgoff and Tgon) throughout the text and figures.

(3). Line 166: Correct "fndings" to "findings". Line 167: Correct "classifed" to "classified".

**Reply:** We apologize for these oversights. All corrections have been made as follows:

(1). Table 1: “Follicula” has been corrected to “Follicular”.

(2). Thyroglobulin state abbreviations: all inconsistent forms (including the typo “Tgofr”) have been replaced with Tg_off_ and Tg_on_ consistently throughout the text, tables, and figure legends.

(3). Line 166: “fndings” corrected to “findings”. Line 167: “classifed” corrected to “classified”.

All changes are clearly marked with track changes in the revised manuscript. We thank the reviewer for carefully identifying these issues.

We thank the Academic Editor and both reviewers for their time and expertise. The revisions have substantially improved the manuscript. We believe the paper is now suitable for publication in PLOS ONE.

Sincerely,

Authors
