# Supplementary material for: 68Ga-FAPI-04 PET/CT has high diagnostic efficacy in Tg-positive and Tg-negative differentiated thyroid cancer
Source: PLoS One. 2026 Sep 3;21(9):e0355343. doi: 10.1371/journal.pone.0355343 (PMC13541195; doi:10.1371/journal.pone.0355343)
Supplement: Peer review history

# Peer review: Decision Letter 0

**DOI:** 10.1371/journal.pone.0355343.r001

**Authors:** Francesco Dondi

2 Jun 2026

PONE-D-26-18193

68Ga-FAPI-04 PET/CT has high diagnostic efficacy in Tg-positive and Tg-negative differentiated thyroid cancer

PLOS One

Dear Dr. wang,

Thank you for submitting your manuscript to PLOS ONE. After careful consideration, we feel that it has merit but does not fully meet PLOS ONE’s publication criteria as it currently stands. Therefore, we invite you to submit a revised version of the manuscript that addresses the points raised during the review process.

Please submit your revised manuscript by Jul 17 2026 11:59PM. If you will need more time than this to complete your revisions, please reply to this message or contact the journal office at plosone@plos.org. When you're ready to submit your revision, log on to https://www.editorialmanager.com/pone/ and select the 'Submissions Needing Revision' folder to locate your manuscript file.

Please include the following items when submitting your revised manuscript:

- A letter that responds to each point raised by the academic editor and reviewer(s). You should upload this letter as a separate file labeled 'Response to Reviewers'.
- A marked-up copy of your manuscript that highlights changes made to the original version. You should upload this as a separate file labeled 'Revised Manuscript with Track Changes'.
- An unmarked version of your revised paper without tracked changes. You should upload this as a separate file labeled 'Manuscript'.

If you would like to make changes to your financial disclosure, please include your updated statement in your cover letter. Guidelines for resubmitting your figure files are available below the reviewer comments at the end of this letter.

If applicable, we recommend that you deposit your laboratory protocols in protocols.io to enhance the reproducibility of your results. Protocols.io assigns your protocol its own identifier (DOI) so that it can be cited independently in the future. For instructions see: https://journals.plos.org/plosone/s/submission-guidelines#loc-laboratory-protocols. Additionally, PLOS ONE offers an option for publishing peer-reviewed Lab Protocol articles, which describe protocols hosted on protocols.io. Read more information on sharing protocols at https://plos.org/protocols?utm_medium=editorial-email&utm_source=authorletters&utm_campaign=protocols.

As the corresponding author, your ORCID iD is verified in the submission system and will appear in the published article. PLOS supports the use of ORCID, and we encourage all coauthors to register for an ORCID iD and use it as well. Please encourage your coauthors to verify their ORCID iD within the submission system before final acceptance, as unverified ORCID iDs will not appear in the published article. *Only*  the individual author can complete the verification step; PLOS staff *cannot*  verify ORCID iDs on behalf of authors.

We look forward to receiving your revised manuscript.

Kind regards,

Francesco Dondi

Academic Editor

PLOS One

Journal Requirements:

When submitting your revision, we need you to address these additional requirements.

1. Please ensure that your manuscript meets PLOS ONE's style requirements, including those for file naming. The PLOS ONE style templates can be found at

https://journals.plos.org/plosone/s/file?id=wjVg/PLOSOne_formatting_sample_main_body.pdf and

https://journals.plos.org/plosone/s/file?id=ba62/PLOSOne_formatting_sample_title_authors_affiliations.pdf

2. PLOS requires an ORCID iD for the corresponding author in Editorial Manager on papers submitted after December 6th, 2016. Please ensure that you have an ORCID iD and that it is validated in Editorial Manager. To do this, go to ‘Update my Information’ (in the upper left-hand corner of the main menu), and click on the Fetch/Validate link next to the ORCID field. This will take you to the ORCID site and allow you to create a new iD or authenticate a pre-existing iD in Editorial Manager.

3. Thank you for stating the following financial disclosure:

“This work was supported by the Youth Fund of Southwest Medical University (Grant No. 2021ZKQN054 and Grant No. 2022QN055 ).”

Please state what role the funders took in the study.  If the funders had no role, please state: "The funders had no role in study design, data collection and analysis, decision to publish, or preparation of the manuscript."

If this statement is not correct you must amend it as needed.

Please include this amended Role of Funder statement in your cover letter; we will change the online submission form on your behalf.

4. In this instance it seems there may be acceptable restrictions in place that prevent the public sharing of your minimal data. However, in line with our goal of ensuring long-term data availability to all interested researchers, PLOS’ Data Policy states that authors cannot be the sole named individuals responsible for ensuring data access (http://journals.plos.org/plosone/s/data-availability#loc-acceptable-data-sharing-methods).

Data requests to a non-author institutional point of contact, such as a data access or ethics committee, helps guarantee long term stability and availability of data. Providing interested researchers with a durable point of contact ensures data will be accessible even if an author changes email addresses, institutions, or becomes unavailable to answer requests.

Before we proceed with your manuscript, please also provide non-author contact information (phone/email/hyperlink) for a data access committee, ethics committee, or other institutional body to which data requests may be sent. If no institutional body is available to respond to requests for your minimal data, please consider if there any institutional representatives who did not collaborate in the study, and are not listed as authors on the manuscript, who would be able to hold the data and respond to external requests for data access? If so, please provide their contact information (i.e., email address). Please also provide details on how you will ensure persistent or long-term data storage and availability.

5. If the reviewer comments include a recommendation to cite specific previously published works, please review and evaluate these publications to determine whether they are relevant and should be cited. There is no requirement to cite these works unless the editor has indicated otherwise.

[Note: HTML markup is below. Please do not edit.]

Reviewers' comments:

Reviewer's Responses to Questions

**Comments to the Author**

1. Is the manuscript technically sound, and do the data support the conclusions?

The manuscript must describe a technically sound piece of scientific research with data that supports the conclusions. Experiments must have been conducted rigorously, with appropriate controls, replication, and sample sizes. The conclusions must be drawn appropriately based on the data presented.

Reviewer #1: Yes

Reviewer #2: Partly

**********

2. Has the statistical analysis been performed appropriately and rigorously?

Reviewer #1: Yes

Reviewer #2: No

**********

3. Have the authors made all data underlying the findings in their manuscript fully available?

The PLOS Data policy requires authors to make all data underlying the findings described in their manuscript fully available without restriction, with rare exception (please refer to the Data Availability Statement in the manuscript PDF file). The data should be provided as part of the manuscript or its supporting information, or deposited to a public repository. For example, in addition to summary statistics, the data points behind means, medians and variance measures should be available. If there are restrictions on publicly sharing data—e.g. participant privacy or use of data from a third party—those must be specified.

Reviewer #1: Yes

Reviewer #2: Yes

**********

4. Is the manuscript presented in an intelligible fashion and written in standard English?

PLOS ONE does not copyedit accepted manuscripts, so the language in submitted articles must be clear, correct, and unambiguous. Any typographical or grammatical errors should be corrected at revision, so please note any specific errors here.

Reviewer #1: Yes

Reviewer #2: Yes

**********

5. Review Comments to the Author

Please use the space provided to explain your answers to the questions above. You may also include additional comments for the author, including concerns about dual publication, research ethics, or publication ethics. (Please upload your review as an attachment if it exceeds 20,000 characters)

Reviewer #1: This retrospective study aimed to evaluate the diagnostic performance of 68Ga-FAPI-04 PET/CT in 147 patients with differentiated thyroid cancer (DTC) after surgery and 131I therapy. The authors compare this emerging imaging modality specifically with the clinical standard of serum thyroglobulin (Tg) testing in different thyrotropin (TSH) states. Strengthens: Clinical Utility in Tg-Negative Cases: The most significant contribution is the identification of metastatic lesions in eight Tg-negative patients, primarily in lymph nodes. This addresses a critical "blind spot" in standard DTC follow-up where low Tg levels might provide a false sense of security. Performance Across TSH States: The data demonstrates that 68Ga-FAPI-04 maintains high accuracy in both TSH-suppressed (95.96%) and TSH-stimulated (100%) states. This suggests patients may not need to undergo the discomfort of levothyroxine withdrawal to achieve high-quality imaging. Quantitative Threshold Optimization: The authors performed a sophisticated analysis of Target-to-Background Ratios (TBR). Identifying the mediastinum as the optimal background reference and establishing a threshold of 1.428 provides clinicians with actionable quantitative criteria for interpreting these scans. Comparison to Standard of Care: By directly comparing PET/CT metrics against Tg sensitivity and specificity, the study frames the new modality within the context of current clinical workflows. Limitations: Retrospective Design and Potential Bias: As a retrospective study, there is inherent selection bias. Furthermore, only 29 of the 147 participants had their PET/CT findings confirmed via histopathology; the remainder relied on clinical follow-up, which is less definitive. The "Inflammation" Hurdle: Consistent with 68Ga-FAPI's known limitations, the study reported false positives in lymph nodes due to inflammatory changes. While the authors addressed this through TBR optimization, the risk of "over-calling" reactive nodes remains a concern for surgical planning. Lack of Direct Comparison with 18F-FDG: Although the authors cite literature comparing FAPI to FDG, this specific cohort was not tested head-to-head. Given that FDG is the current alternative for Tg-positive/Iodine-negative (TENIS) cases, a direct comparison would have strengthened the argument for switching to FAPI. The authors should make clear in the introduction that ⁶⁸Ga-FAPI may be useful for TENIS or for any state of DTC. Please note that the quality of images in the manuscript is poor, so the reviewer could not see lesions clearly.

Reviewer #2: This retrospective study examines the diagnostic efficacy of 68Ga-FAPI-04 PET/CT in patients with differentiated thyroid carcinoma (DTC) following 131I therapy, with a particular focus on its clinical value in localizing lesions among thyroglobulin (Tg)-negative patients. The inclusion of 147 patients (99 TSH-suppressed, 48 TSH-stimulated) provides a robust sample size for assessing an emerging radiotracer. The quantitative analysis, specifically determining a target-to-background ratio (TBR) threshold of 1.428 utilizing the mediastinum as a reference, is a notable contribution that could aid in standardizing image interpretation. However, several methodological and statistical limitations regarding the reference standard and threshold validation must be addressed before the manuscript can be considered for publication.

Major Comments

1. The study reports that only 29 of the 147 patients (19.7%) underwent histopathological biopsy as the definitive reference standard. For the remaining patients, serial serum Tg monitoring over a minimum of one year was utilized alongside imaging to confirm disease status. Because the primary objective of this study is to compare the diagnostic accuracy of 68Ga-FAPI-04 PET/CT directly against Tg testing, utilizing Tg as the ground truth to subsequently calculate the sensitivity and specificity of Tg creates a significant incorporation bias.

Recommendation: The authors should perform a subgroup analysis restricting the diagnostic performance comparison (FAPI vs. Tg) exclusively to the 29 patients with biopsy-proven results. Furthermore, the median follow-up time for the clinically monitored cohort should be explicitly stated, and this bias must be discussed in the Limitations section.

2. The study derives an optimal TBR threshold of 1.428 (AUC = 0.982) for predicting lymph node metastases using the entire patient cohort. Deriving a diagnostic cutoff and reporting its performance metrics on the exact same dataset carries a high risk of statistical overfitting.

Recommendation: To strengthen the reliability of this threshold, the authors should perform an internal validation (e.g., K-fold cross-validation or bootstrapping with 1,000 resamples) and report the validated accuracy. If internal validation is not feasible, the lack of an independent validation cohort must be explicitly stated as a major limitation in the Discussion.

3. The positive 68Ga-FAPI-04 scan rate in this cohort is relatively high at 38.8% (57/147). This suggests that the enrolled population may inherently possess a higher pre-test probability for recurrence or metastasis than a standard post-therapy follow-up population.

Recommendation: Please clarify the specific clinical indications for ordering the 68Ga-FAPI-04 imaging in the Methods section (e.g., were patients scanned due to suspected recurrence on ultrasound, or as a routine pre-therapy assessment?). The authors should also discuss how this potential selection bias might influence the reported Positive Predictive Value (PPV) and Negative Predictive Value (NPV).

4. The manuscript frequently references the limitations of 18F-FDG PET/CT and cites literature suggesting FAPI's superiority in DTC. However, the current study does not provide direct comparative data.

Recommendation: If a subset of the enrolled patients underwent 18F-FDG PET/CT within a similar timeframe, incorporating a head-to-head comparative analysis would significantly elevate the manuscript's impact. If such data is entirely unavailable, the authors should soften claims regarding comparative superiority and list the absence of 18F-FDG data as a limitation.

5. There are some spelling mistakes and inconsistent formats in the original manuscript.

(1). Table 1: Under the "Pathologic subtype" category, the term "Follicula" is misspelled and should be corrected to "Follicular".

(2). The manuscript uses inconsistent formatting and abbreviations for thyroglobulin states, interchanging Tgoff, Tgofr, and Tgoff. Please rigorously standardize these terms (e.g., consistently using Tgoff and Tgon) throughout the text and figures.

(3). Line 166: Correct "fndings" to "findings". Line 167: Correct "classifed" to "classified".

**********

6. PLOS authors have the option to publish the peer review history of their article (what does this mean?). If published, this will include your full peer review and any attached files.

If you choose “no”, your identity will remain anonymous but your review may still be made public.

**Do you want your identity to be public for this peer review?**  For information about this choice, including consent withdrawal, please see our Privacy Policy.

Reviewer #1: **Yes:**  Mai Hong Son

Reviewer #2: No

**********

[NOTE: If reviewer comments were submitted as an attachment file, they will be attached to this email and accessible via the submission site. Please log into your account, locate the manuscript record, and check for the action link "View Attachments". If this link does not appear, there are no attachment files.]

To ensure your figures meet our technical requirements, please review our figure guidelines: https://journals.plos.org/plosone/s/figures

You may also use PLOS’s free figure tool, NAAS, to help you prepare publication quality figures: https://journals.plos.org/plosone/s/figures#loc-tools-for-figure-preparation.

NAAS will assess whether your figures meet our technical requirements by comparing each figure against our figure specifications.

# Author comment: Author response to Decision Letter 1

**DOI:** 10.1371/journal.pone.0355343.r002

17 Jul 2026

Dear Academic Editor Dr. Francesco Dondi and Reviewers,

Thank you for your thoughtful comments and valuable suggestions on our manuscript (PONE-D-26-18193). We have carefully addressed all points raised by the editor and reviewers. Below we provide point-by-point responses. All changes in the revised manuscript are marked using track changes.

Response to Academic Editor

1. Please ensure that your manuscript meets PLOS ONE's style requirements, including those for file naming.

Reply: We have revised the manuscript to meet PLOS ONE’s formatting guidelines.

2. PLOS requires an ORCID iD for the corresponding author in Editorial Manager on papers submitted after December 6th, 2016. Please ensure that you have an ORCID iD and that it is validated in Editorial Manager. To do this, go to ‘Update my Information’ (in the upper left-hand corner of the main menu), and click on the Fetch/Validate link next to the ORCID field. This will take you to the ORCID site and allow you to create a new iD or authenticate a pre-existing iD in Editorial Manager.

Reply: The corresponding author’s ORCID iD has been verified in Editorial Manager.

3. Thank you for stating the following financial disclosure:“This work was supported by the Youth Fund of Southwest Medical University (Grant No. 2021ZKQN054 and Grant No. 2022QN055 ).” Please state what role the funders took in the study. If the funders had no role, please state: "The funders had no role in study design, data collection and analysis, decision to publish, or preparation of the manuscript." If this statement is not correct you must amend it as needed. Please include this amended Role of Funder statement in your cover letter; we will change the online submission form on your behalf.

Reply: We have added the following statement in the cover letter: “This work was supported by the Youth Fund of Southwest Medical University (Grant No. 2021ZKQN054 and Grant No. 2022QN055). The funders had no role in study design, data collection and analysis, decision to publish, or preparation of the manuscript. The funding was received by the first author (Lixian Mou, Grant No. 2021ZKQN054) and the corresponding author (Wei Wang, Grant No. 2022QN055), who contributed to study design, data analysis, and manuscript writing as co‑authors.”

4. In this instance it seems there may be acceptable restrictions in place that prevent the public sharing of your minimal data. However, in line with our goal of ensuring long-term data availability to all interested researchers, PLOS’ Data Policy states that authors cannot be the sole named individuals responsible for ensuring data access (http://journals.plos.org/plosone/s/data-availability#loc-acceptable-data-sharing-methods). Data requests to a non-author institutional point of contact, such as a data access or ethics committee, helps guarantee long term stability and availability of data. Providing interested researchers with a durable point of contact ensures data will be accessible even if an author changes email addresses, institutions, or becomes unavailable to answer requests. Before we proceed with your manuscript, please also provide non-author contact information (phone/email/hyperlink) for a data access committee, ethics committee, or other institutional body to which data requests may be sent. If no institutional body is available to respond to requests for your minimal data, please consider if there any institutional representatives who did not collaborate in the study, and are not listed as authors on the manuscript, who would be able to hold the data and respond to external requests for data access? If so, please provide their contact information (i.e., email address). Please also provide details on how you will ensure persistent or long-term data storage and availability.

Reply: Due to ethical restrictions (participant consent and institutional review board approval), data cannot be publicly shared. Requests for access to the minimal anonymized data set may be directed to the Institutional Data Access / Ethics Committee of the Affiliated Hospital of Southwest Medical University (Contact: Shumao Zhang, Email: 540950842@qq.com, Phone: 0830-3165721). Data will be stored on the hospital's secure server for at least 10 years.

5. If the reviewer comments include a recommendation to cite specific previously published works, please review and evaluate these publications to determine whether they are relevant and should be cited. There is no requirement to cite these works unless the editor has indicated otherwise.

Reply: No specific works were recommended for citation. We have uploaded the Response to Reviewers, the revised manuscript with track changes, and a clean manuscript.

Response to Reviewer #1

We thank Reviewer #1 for the positive evaluation and constructive comments on our manuscript. We have carefully addressed all the concerns raised. All changes are marked with track changes in the revised manuscript.

Comment 1: Retrospective Design and Potential Bias: As a retrospective study, there is inherent selection bias. Furthermore, only 29 of the 147 participants had their PET/CT findings confirmed via histopathology; the remainder relied on clinical follow‑up, which is less definitive.

Reply: We acknowledge these limitations. To address the potential incorporation bias due to limited histopathological confirmation, we performed a subgroup analysis restricted to the 29 biopsy‑proven patients (S1 Table). In this subgroup, the sensitivity of 68Ga‑FAPI‑04 PET/CT remained high (94.12-100%), consistent with the full‑cohort analysis. For the 118 clinically followed patients, the median follow‑up duration was 18.3 months (IQR: 14.2-24.1 months), which we believe is sufficient to validate most PET/CT findings. We have explicitly discussed these limitations in the Discussion section.

Comment 2: The "Inflammation" Hurdle: Consistent with 68Ga‑FAPI’s known limitations, the study reported false positives in lymph nodes due to inflammatory changes. While the authors addressed this through TBR optimization, the risk of 'over‑calling' reactive nodes remains a concern for surgical planning.

Reply: We agree with the reviewer that inflammatory false positives remain a challenge for FAPI PET/CT. We have revised the Discussion to explicitly address this limitation. Specifically, we now emphasize that FAPI PET/CT findings should be correlated with conventional imaging (ultrasonography) and, when clinically warranted, verified by cytology or histopathology before surgical decision‑making. This multimodal approach is essential to avoid over‑interpretation and ensure appropriate patient management. All changes are marked in the revised manuscript.

Comment 3: Lack of Direct Comparison with 18F-FDG: Although the authors cite literature comparing FAPI to FDG, this specific cohort was not tested head‑to‑head. Given that FDG is the current alternative for Tg‑positive/Iodine‑negative (TENIS) cases, a direct comparison would have strengthened the argument for switching to FAPI.

Reply: We acknowledge the reviewer’s concern. Only few patients in our cohort underwent 18F‑FDG PET/CT within a comparable timeframe, which is an insufficient sample size for a meaningful head‑to‑head analysis. Therefore, we have: (1) softened all claims of superiority over FDG throughout the manuscript; and (2) explicitly added the lack of FDG comparison as a limitation in the Discussion section. We agree that future prospective paired studies are needed to determine the optimal imaging strategy.

Comment 4: The authors should make clear in the introduction that 68Ga‑FAPI may be useful for TENIS or for any state of DTC.

Reply: We agree with the reviewer and have revised the Introduction accordingly. The following sentence has been added (lines 102-104 in manuscript_revised): “Therefore, 68Ga‑FAPI‑04 PET/CT may be useful not only in patients with TENIS syndrome but also in various states of DTC, including both Tg‑positive and Tg‑negative conditions, regardless of TSH stimulation status.”

Comment 5: Please note that the quality of images in the manuscript is poor, so the reviewer could not see lesions clearly.

Reply: We apologize for the poor image quality in the original submission. To comprehensively address this issue, we have taken the following measures:

(1) Added corresponding transverse PET/CT fusion images (A’-H’) in Fig 1 to provide precise anatomical localization of each lesion.

(2) Converted all figures to TIFF format with LZW compression at 600 dpi, following PLOS ONE's specifications, and verified them using the NAAS quality check tool.

Response to Reviewer #2

We thank Reviewer #2 for the thorough and rigorous review.

Major Comments

Comment 1: The study reports that only 29 of the 147 patients (19.7%) underwent histopathological biopsy as the definitive reference standard. For the remaining patients, serial serum Tg monitoring over a minimum of one year was utilized alongside imaging to confirm disease status. Because the primary objective of this study is to compare the diagnostic accuracy of 68Ga-FAPI-04 PET/CT directly against Tg testing, utilizing Tg as the ground truth to subsequently calculate the sensitivity and specificity of Tg creates a significant incorporation bias. Recommendation: The authors should perform a subgroup analysis restricting the diagnostic performance comparison (FAPI vs. Tg) exclusively to the 29 patients with biopsy-proven results. Furthermore, the median follow-up time for the clinically monitored cohort should be explicitly stated, and this bias must be discussed in the Limitations section.

Reply: We thank the reviewer for this important comment. We have performed the requested subgroup analysis on the 29 biopsy‑confirmed patients (S1 Table) and added the median follow‑up time (18.3 months, IQR 14.2–24.1 months) in the Methods and Results sections. We have also expanded the Limitations paragraph to discuss incorporation bias. As shown in S1 Table, the sensitivity of 68Ga‑FAPI‑04 PET/CT remained high in both TSH states (100% in TSHon, 94.1% in TSHoff), consistent with the full‑cohort analysis (Table 2). The extreme values (N/A and 0%) in this subgroup are explained by two factors: (i) In clinical practice, biopsy is performed only when recurrence is suspected, so a biopsy‑confirmed cohort naturally lacks true‑negative patients; (ii) All three false‑positive (inflammatory lymph nodes, Figure 2G,2K,2O) and the single false‑negative (a lymph node, Figure 2D) findings of the entire study were concentrated in this small subgroup. Therefore, the 0% specificity and 0% NPV do not indicate poor diagnostic performance of FAPI PET/CT. The full‑cohort analysis (Table 2) demonstrates excellent specificity (95.31% for TSHoff) and NPV (98.39% for TSHoff). We have added a detailed discussion of these points in the Results and Discussion sections (see revised manuscript). We believe the incorporation bias does not undermine our main conclusions.

Comment 2: The study derives an optimal TBR threshold of 1.428 (AUC = 0.982) for predicting lymph node metastases using the entire patient cohort. Deriving a diagnostic cutoff and reporting its performance metrics on the exact same dataset carries a high risk of statistical overfitting. Recommendation: To strengthen the reliability of this threshold, the authors should perform an internal validation (e.g., K-fold cross-validation or bootstrapping with 1,000 resamples) and report the validated accuracy. If internal validation is not feasible, the lack of an independent validation cohort must be explicitly stated as a major limitation in the Discussion.

Reply: We thank the reviewer for this important statistical concern. To address potential overfitting, we performed a 10‑fold cross‑validation. The mean cross‑validated AUC was 0.974 (95% CI: 0.944-1.000; range: 0.836-1.000), with corresponding mean sensitivity of 0.980 and mean specificity of 0.972. The mean AUC was highly consistent with the original value (0.982), confirming that the TBR threshold is robust and not overfitted to our dataset. We have added the cross‑validation methodology to the Statistical Analysis section, the results to the Results section, and a corresponding limitation in the Discussion section. A new S1 Fig showing the cross‑validation ROC curves has also been provided. At the same time, we acknowledge that our findings are derived from a single‑center cohort and lack external validation, which has been explicitly stated as a limitation in the Discussion. Future studies with larger, independent, and preferably multicenter cohorts are warranted to further validate the generalizability of our model.

Comment 3: The positive 68Ga-FAPI-04 scan rate in this cohort is relatively high at 38.8% (57/147). This suggests that the enrolled population may inherently possess a higher pre-test probability for recurrence or metastasis than a standard post-therapy follow-up population. Recommendation: Please clarify the specific clinical indications for ordering the 68Ga-FAPI-04 imaging in the Methods section (e.g., were patients scanned due to suspected recurrence on ultrasound, or as a routine pre-therapy assessment?). The authors should also discuss how this potential selection bias might influence the reported Positive Predictive Value (PPV) and Negative Predictive Value (NPV).

Reply: We thank the reviewer for this important observation. We agree that the 38.78% positive scan rate in our cohort indicates a higher pre‑test probability than an unselected DTC follow‑up population. To address this, we have taken the following steps: In the Methods section (Inclusion criteria), we have explicitly defined the clinical indications for performing 68Ga‑FAPI‑04 PET/CT, categorizing them into four groups. In the Results section (Patient characteristics), we have reported the distribution of these indications (Tg elevation with negative imaging, suspicious imaging findings, physician‑judged high‑risk restaging, and routine follow‑up or other reasons). In the Discussion section (Limitations), we have added a specific limitation (Second) acknowledging that this selection bias may overestimate PPV and underestimate NPV, and that prospective studies in consecutive unselected patients are warranted.

Comment 4: The manuscript frequently references the limitations of 18F-FDG PET/CT and cites literature suggesting FAPI's superiority in DTC. However, the current study does not provide direct comparative data. Recommendation: If a subset of the enrolled patients underwent 18F-FDG PET/CT within a similar timeframe, incorporating a head-to-head comparative analysis would significantly elevate the manuscript's impact. If such data is entirely unavailable, the authors should soften claims regarding comparative superiority and list the absence of 18F-FDG data as a limitation.

Reply: We thank the reviewer for this suggestion. Since only few patients had 18F-FDG PET/CT within a comparable timeframe (insufficient for analysis), we have:

(1). Softened all claims of superiority over 18F-FDG. In the Introduction, “superior sensitivity” was changed to “may have higher sensitivity”. In the Discussion, we removed the detailed criticism of 18F-FDG limitations and now state that 68Ga-FAPI-04 “may serve as a complementary tool” to 18F-FDG.

(2). Added the lack of 18F-FDG comparison as a limitation. The revised limitation (now “Third”) reads: “Third, this study did not include a head‑to‑head comparison with 18F‑FDG PET/CT, Although previous studies have suggested potential advantages of 68Ga-FAPI-04 over 18F-FDG PET/CT in DTC, the absence of direct comparative data in our cohort limits any claims of superiority. Future prospective studies with paired 18F‑FDG and 68Ga-FAPI-04 imaging are needed to determine the optimal imaging strategy”. All changes are marked in the revised manuscript.

Comment 5: There are some spelling mistakes and inconsistent formats in the original manuscript.

(1). Table 1: Under the "Pathologic subtype" category, the term "Follicula" is misspelled and should be corrected to "Follicular".

(2). The manuscript uses inconsistent formatting and abbreviations for thyroglobulin states, interchanging Tgoff, Tgofr, and Tgoff. Please rigorously standardize these terms (e.g.

Attachment Submitted filename: Response to Reviewers.docx

# Editor report: Decision Letter 1

**DOI:** 10.1371/journal.pone.0355343.r003

**Authors:** Francesco Dondi

20 Jul 2026

68Ga-FAPI-04 PET/CT has high diagnostic efficacy in Tg-positive and Tg-negative differentiated thyroid cancer

PONE-D-26-18193R1

Dear Dr. wang,

We’re pleased to inform you that your manuscript has been judged scientifically suitable for publication and will be formally accepted for publication once it meets all outstanding technical requirements.

Within one week, you’ll receive an e-mail detailing the required amendments. When these have been addressed, you’ll receive a formal acceptance letter and your manuscript will be scheduled for publication.

An invoice will be generated when your article is formally accepted. Please note, if your institution has a publishing partnership with PLOS and your article meets the relevant criteria, all or part of your publication costs will be covered. Please make sure your user information is up-to-date by logging into Editorial Manager at Editorial Manager® and clicking the ‘Update My Information' link at the top of the page. For questions related to billing, please contact billing support.

If your institution or institutions have a press office, please notify them about your upcoming paper to help maximize its impact. If they’ll be preparing press materials, please inform our press team as soon as possible -- no later than 48 hours after receiving the formal acceptance. Your manuscript will remain under strict press embargo until 2 pm Eastern Time on the date of publication. For more information, please contact onepress@plos.org.

Kind regards,

Francesco Dondi

Academic Editor

PLOS One

Additional Editor Comments (optional):

Reviewers' comments:

# Editor report: Acceptance letter

**DOI:** 10.1371/journal.pone.0355343.r004

**Authors:** Francesco Dondi

PONE-D-26-18193R1

PLOS One

Dear Dr. wang,

I'm pleased to inform you that your manuscript has been deemed suitable for publication in PLOS One. Congratulations! Your manuscript is now being handed over to our production team.

At this stage, our production department will prepare your paper for publication. This includes ensuring the following:

* All references, tables, and figures are properly cited

* All relevant supporting information is included in the manuscript submission,

* There are no issues that prevent the paper from being properly typeset

You will receive further instructions from the production team, including instructions on how to review your proof when it is ready. Please keep in mind that we are working through a large volume of accepted articles, so please give us a few days to review your paper and let you know the next and final steps.

Lastly, if your institution or institutions have a press office, please let them know about your upcoming paper now to help maximize its impact. If they'll be preparing press materials, please inform our press team within the next 48 hours. Your manuscript will remain under strict press embargo until 2 pm Eastern Time on the date of publication. For more information, please contact onepress@plos.org.

You will receive an invoice from PLOS for your publication fee after your manuscript has reached the completed accept phase. If you receive an email requesting payment before acceptance or for any other service, this may be a phishing scheme. Learn how to identify phishing emails and protect your accounts at https://explore.plos.org/phishing.

If we can help with anything else, please email us at customercare@plos.org.

Thank you for submitting your work to PLOS ONE and supporting open access.

Kind regards,

PLOS ONE Editorial Office Staff

on behalf of

Dr Francesco Dondi

Academic Editor

PLOS One
